# Congenital Umbilical Hernia1Read before the Buffalo Pathological Society, August 23, 1889.

**Published:** 1890-01

**Authors:** John Parmenter

**Affiliations:** Lecturer on Anatomy, Medical Department University of Buffalo; 372 Franklin Street


### CONGENITAL UMBILICAL HERNIA.[1]

By JOHN PARMENTER, M. D.,

Lecturer on Anatomy, Medical Department University of Buffalo.

In early fetal life, the abdominal wall is widely open in front, and through this opening the greater part of the abdominal viscera pass. Toward the end of the third month this cleft closes, the abdominal organs enter the abdomen, and there remains only an orifice, through which the umbilical vessels pass. Not rarely a knuckle of intestine escapes the retraction of the viscera and remains outside, forming a tumor, covered with amniotic membrane, and known as congenital umbilical hernia. It thus differs essentially from other hernias, in that its contents are organs *which have never been within the abdominal cavity*, and not organs which have been in the abdomen and have escaped from the same, as in the many forms of acquired hernias, occurring at all ages, from the new-born to the aged.

The umbilical cord contains, in addition to the bloodvessels, two other structures, viz., the urachus, a continuation of the bladder and springing from the allantois; and the ductus vitello-intestinalis, a prolongation of the intestine, and arising from the vesiculo-umbilicalis. Outside the abdomen of the six weeks' embryo, normally, are found the most distal coils of the ileum, a part of the cecum, and the vitelline duct. About the tenth week, this latter is torn off, and the intestines, being freed, enter the abdominal cavity. The cleft in which they lay closes. If this separation does not occur, or there is no pulling upon the duct, the intestines will remain outside the abdomen and in the cord. The umbilical opening may become still

1. Read before the Buffalo Pathological Society, August 23, 1889.

greater by the intestines being drawn further out. A time comes, however, when a tendency to contraction is manifested at the opening, as shown by the constriction of resisting structures, like the liver, the part appearing like a club-shaped prolongation of the organ.

The contents of congenital umbilical hernia vary with its size. The smaller contain, perhaps, only a knuckle of small intestine; the larger may have any or all the abdominal organs within them.

As to the etiology, two things are to be noted: on the one hand, a more or less imperfect abdominal wall, and, on the other, viscera, which are outside the abdomen. Authors differ in attaching greater importance to the one than to the other, and, hence, two theories have arisen. One claims this hernia to be due to an arrest of development, the other regards this arrest as consecutive to an adhesive peritonitis in the part of the peritoneum normally enclosed in the cord. It is easy to see how such adhesions could oppose the entrance of the viscera into the abdomen, and prevent obliteration of the umbilical ring. Peritonitis, however, is often wanting, and may occur after the passage inwards of the viscera. A third theory (Scarpa's) has been advanced. This supposes traction on the cord, in intra-uterine life, to be the cause of this hernia. The only case observed by the writer in his own practice lends color to this theory. The cord, not of unusual length, was wound round the child's neck thrice, and caused marked traction upon the umbilicus. Within the protrusion, a small portion of an intestinal loop could be plainly seen. The child recovered from this, dying some months later of entero-colitis. However we may regard these theories concerning the etiology of the affection under consideration, one thing is to be remembered. It is, that, at the time of birth, the umbilical ring is not yet obliterated, and a communication exists between the cavities of the cord and abdomen. Therefore, slight causes, falling down of the mother, blows on the abdomen, manipulations during pressure upon the child's body in difficult confinements, first respiratory efforts and cries, acting singly or together, can cause protrusion of the viscera. Such hernias, however, although called congenital, have anatomical characters and a mode of development quite different from those of true congenital hernias, since, as before stated, the viscera in the latter cases have never been within the abdominal cavity; whereas, in the former, they have once entered and again appeared outside the abdomen.

The coverings of a congenital umbilical hernia are: (1) the elongated skin of the abdomen continued into thin transparent amnion; (2) a thin fibrous prolongation of the connective tissue of the linea alba, occurring at the base of the hernia, and continued into Wharton's jelly; (3) peritoneal prolongation from the anterior abdominal wall; a prolongation of the sac has been noted without coincident rupture (Kleb's) suggesting that when the intestine slips into the abdomen, the peritoneum remains in a way analogous to the processus vaginalis in inguinal hernia. Growth of the body draws the former into the abdomen, while the latter becomes obliterated in its original position. Adhesions between the sac and its contents are common.

Congenital umbilical hernia appears usually as a conical tumor, base to the abdominal wall. Smaller hernias are globular. The external surface is smooth and shiny, the contents of the sac being plainly visible through its transparent walls. The superior semi-circumference only, however, is transparent, the inferior being opaque. The base and remaining portion are separated by a very thick, reddish border which deepens, becoming a furrow as the cord comes nearer falling off. The volume is increased by cries, respiratory and other movements of the child, and the greater the volume the easier its reduction.

Large hernias are easily diagnosticated. Small ones, containing only a knuckle of intestine, require close examination. It is quite probable that ligatures have included intestine which has never been perceived when symptoms of strangulation and death supervene. Fecal fistula may ensue with results dependent upon the portion of intestines involved. Obstetricians should examine carefully every cord before ligation.

The prognosis is obviously bad, over fifty per cent. dying from this affection. Gangrene and peritonitis are the common causes of death. Sometimes hepatic hemorrhage or phlebitis of the umbilical vein is to blame.

Because this hernia occurs once in 2,000 children, and because cases left to nature are so often fatal, radical operation is replacing older methods. Many successful cases are now on record and the list is constantly growing. Kocher, in his masterly essay, "Hernien im Kindesalter," to which the writer is largely indebted, says in this connection :

> In reading through the newest handbooks upon children's diseases, one is easily convinced that individual experiences have exercised a far too unfavorable judgment upon the prognosis of umbilical hernias. Certain authors speak in such a way that the reader must necessarily suppose that in a case of this kind, he has nothing else to do than look on and shake his head. It is on this account to be emphasized that umbilical hernias in their worst forms and complications have shown recoveries, and that the physician must charge himself with a great fault of omission in every case where he has permitted a single one of the very exact indications to go unfulfilled.

Small and easily returnable hernias are treated by reduction, and retaining them then with plaster as usually done in the acquired forms occurring in the new-born. If irreducible, an antiseptic bandage applied until the cord separates will do better than using much force. Expectant treatment is commendable, because it has given excellent results and can always be applied. Herniotomy is applicable in only two classes of cases, according to Kocher; those in which strangulation has occurred, and those which are pear-shaped with the small end toward the abdominal wall; these latter contain only intestine and are very prone to strangulate.

372 FRANKLIN STREET.

